# Supplementary material for: SMQVP: A Web Application for Spatial Metabolomics Quality Visualization and Processing
Source: Metabolites. 2025 May 27;15(6):354. doi: 10.3390/metabo15060354 (PMC12195481; doi:10.3390/metabo15060354)

SMQVP: a web application for spatial metabolomics quality visualization and processing

Zhanlong Mei[1], Wan Sun[1], Haoke Deng[1], Yun Zhao[1], Xiaolian Ning[2], Chunlu Feng[1], Jin Zi[1]*

1 BGI Genomics, Shenzhen, China.

2 BGI Research, Shenzhen, China

Supplementary Table 1. Data quality dimensions and their corresponding Quality Visualization Points (QVPs)

Supplementary Table 2. Comparative analysis of SMQVP and existing spatial metabolomics tools.

Supplementary Table 3. Computational performance of SMQVP across datasets of varying scales.

Figure S1. The spatial intensity distribution map of choline (104.1069)

Figure S2. Screenshot of manual selection of tissue and background regions: A region is selected using the lasso tool and then designated as either a tissue or background region.

Figure S3. Identification of background pixels. Hematoxylin and Eosin (H&E) staining image (A); Screenshot of QVP3: the proportion of the tissue enriched ions (B); The total intensity image constructed with the tissue-enriched ions (C); Screenshot of background pixel identification: By setting a threshold, the pixels in the image are classified as either background or tissue regions (D).

Figure S4. The spatial distribution of ions with the noise scores close to 20.

Figure S5. The spatial distribution of ions with the noise scores close to 60.

Figure S6. The spatial distribution of ions with the noise scores close to 90.

Figure S7. Screenshot depicting the QVP1 visualization of the AP-SMALDI dataset.

Figure S8. Screenshot depicting the QVP2 visualization of the AP-SMALDI dataset.

Figure S9. Screenshot depicting the QVP3 visualization of the AP-SMALDI dataset.

Figure S10. Screenshot depicting the QVP4 visualization of the AP-SMALDI dataset.

Figure S11. Screenshot depicting the QVP5 visualization of the AP-SMALDI dataset.

Figure 12. Screenshot depicting the QVP6 visualization of the AP-SMALDI dataset.

Figure S13. Screenshot depicting the QVP7 visualization of the AP-SMALDI dataset.

Figure S14. Screenshot depicting the QVP8 visualization of the AP-SMALDI dataset.

Supplementary Table 1. Data quality dimensions and their corresponding Quality Visualization Points (QVPs)

**Category**	**Subcategory**	**Corresponding QVP**
**Contamination Detection**	Contaminant Peak Detection (e.g., polymers)	QVP1: Spectral Analysis of Background and Tissue Regions
**Overall Data Quality**	Signal Stability during Data Acquisition	QVP2: Background Region Consistency
	Overall Signal Intensity	QVP5: Analysis of Intensity Distribution
	Overall Missing Rate	QVP6: Assessment of Missing Value Distribution
**Ion Credibility**	High Ion Intensity in Tissue Regions	QVP3: Proportion of Tissue-Enriched Ions
	Proportion of Noise Ions	QVP4: Identification and Proportion of Noise Ions
	Proportion of Ions with Isotopic Peaks	QVP7: Isotopic Peak Ratio Assessment
	Proportion of Ions for which Adduct Forms Can Be Calculated	QVP8: Adduct Ion Ratio Assessment

Supplementary Table 2. Comparative analysis of SMQVP and existing spatial metabolomics tools.

**Feature**	**SMQVP**	**Cardinal** [1]	**MSIReader** [2]	**MassImager** [3]	**multi-MSIProcessor** [4]
**Background Region Analysis**	✔ Spectral comparison, Pearson correlation (QVP1, QVP2)	✘	✘	✘	✘
**Tissue-Enriched Ion Identification**	✔ Wilcoxon rank-sum test, fold-change (QVP3)	✘	✘	✘	✘
**Noise Ion Filtering**	✔ Quadrat test for spatial randomness (QVP4)	✘	✘	✘	✘
**Ion intensity and missing Value Assessment**	✔ Pixel/ion missing ratios, spatial visualization (QVP6)	✔	✔	✔	✔
**Isotopic Peak Identification**	✔ MetaboCoreUtils + spatial correlation (QVP7)	✘	✔	✔	✘
**Adduct Ion Analysis**	✔ Customizable adduct lists + spatial correlation (QVP8)	✘	✔	✘	✘
**Interactive Visualization**	✔ Shiny-based GUI with real-time adjustments	✘	✔	✔	✔
**Open Source**	✔ GitHub	✔	✘	✘	✔

References:

1. Bemis, K.D.; Harry, A.; Eberlin, L.S.; Ferreira, C.; van de Ven, S.M.; Mallick, P.; Stolowitz, M.; Vitek, O. Cardinal: An R Package for Statistical Analysis of Mass Spectrometry-Based Imaging Experiments. *Bioinformatics* **2015**, *31*, 2418–2420, doi:10.1093/bioinformatics/btv146.

2. Bokhart, M.T.; Nazari, M.; Garrard, K.P.; Muddiman, D.C. MSiReader v1.0: Evolving Open-Source Mass Spectrometry Imaging Software for Targeted and Untargeted Analyses. *J Am Soc Mass Spectrom* **2018**, *29*, 8–16, doi:10.1007/s13361-017-1809-6.

3. He, J.; Huang, L.; Tian, R.; Li, T.; Sun, C.; Song, X.; Lv, Y.; Luo, Z.; Li, X.; Abliz, Z. MassImager: A Software for Interactive and in-Depth Analysis of Mass Spectrometry Imaging Data. *Anal Chim Acta* **2018**, *1015*, 50–57, doi:https://doi.org/10.1016/j.aca.2018.02.030.

4. Bi, S.; Wang, M.; Pu, Q.; Yang, J.; Jiang, N.; Zhao, X.; Qiu, S.; Liu, R.; Xu, R.; Li, X.; et al. Multi-MSIProcessor: Data Visualizing and Analysis Software for Spatial Metabolomics Research. *Anal Chem* **2024**, *96*, 339–346, doi:10.1021/acs.analchem.3c04192.

Supplementary Table 3. Computational performance of SMQVP across datasets of varying scales.

**Dataset**	**Mouse Brain (Low-res)**	**Mouse Brain (Demo)**	**Human Stomach (High-res)**
**Spatial Resolution/µm**	100	50	20
**Pixel Number**	21,164	53,812	118,604
**Ion Number**	2,843	2,654	2,898
**Raw data size/GB**	8.8	20.8	77
**Peak table size/MB**	298	714.3	1700
**Average Memory Usage/GB**	4.27	5.1	10.47
**Runtime/min**	3	6	25

Figure S1. The spatial intensity distribution map of choline (104.1069)

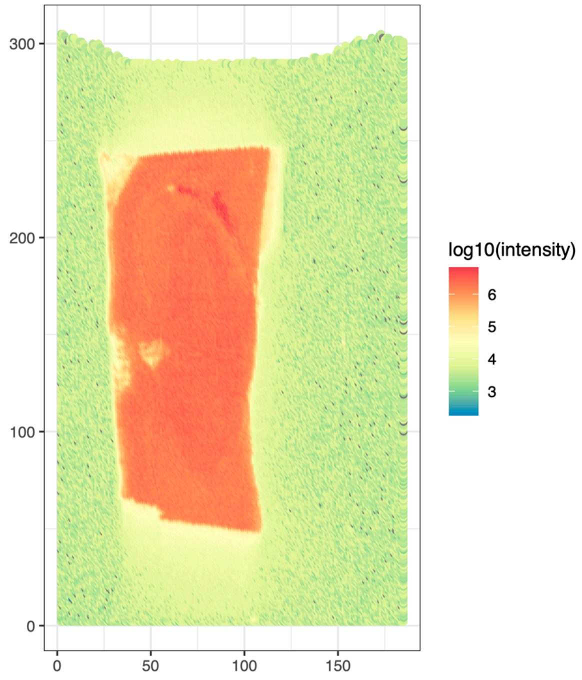

Figure S2. Manual selection of tissue and background regions. A region is manually selected using the lasso tool within the SMQVP interface and subsequently designated as either a tissue or background region via dedicated buttons. The final visualization depicts the manually selected tissue regions colored green and background regions colored orange.

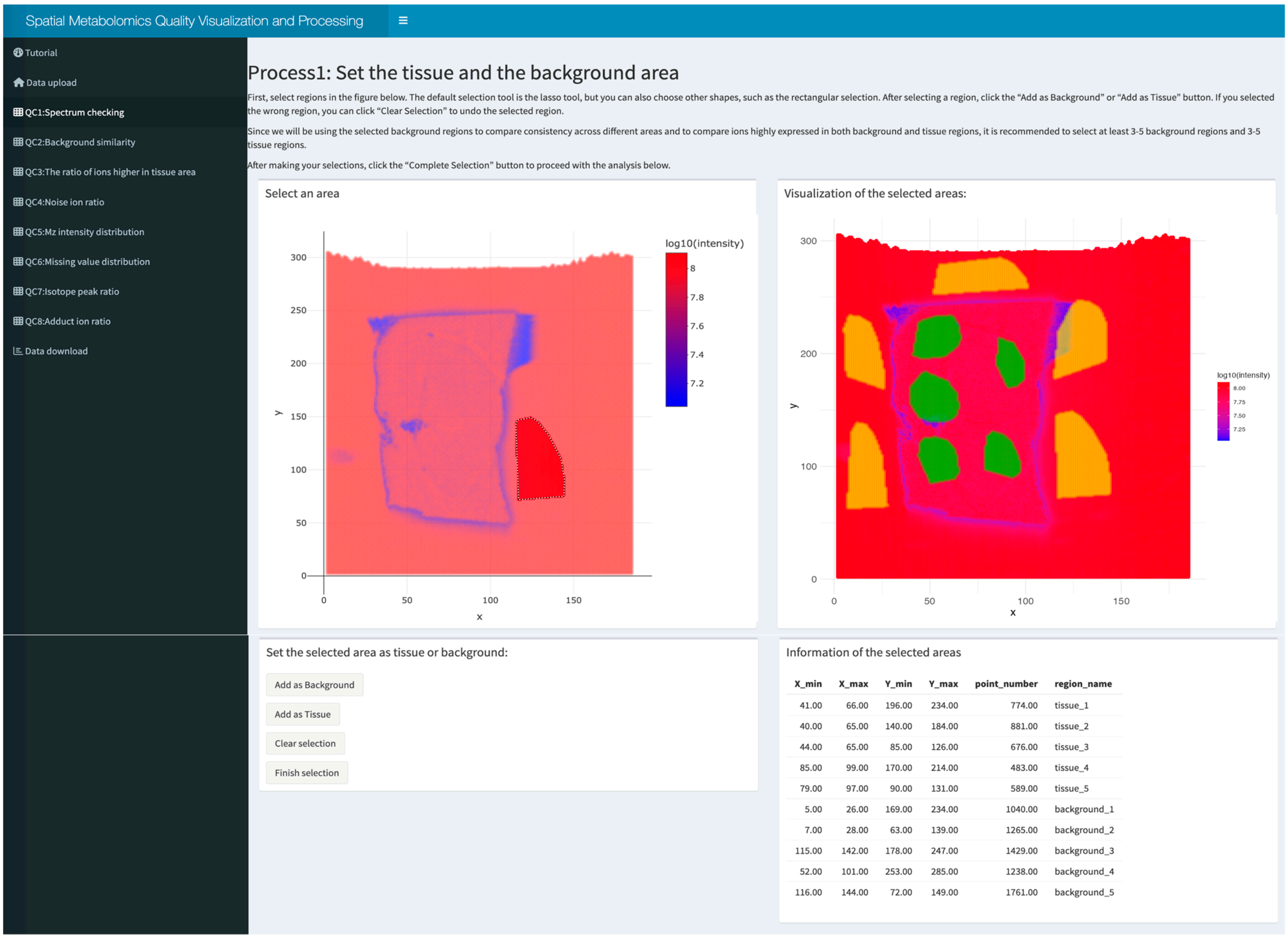

Figure S3. Identification of background pixels. Hematoxylin and Eosin (H&E) staining image (A); Screenshot of QVP3: the proportion of the tissue enriched ions (B); The total intensity image constructed with the tissue-enriched ions (C); Screenshot of background pixel identification: By setting a threshold, the pixels in the image are classified as either background or tissue regions (D).

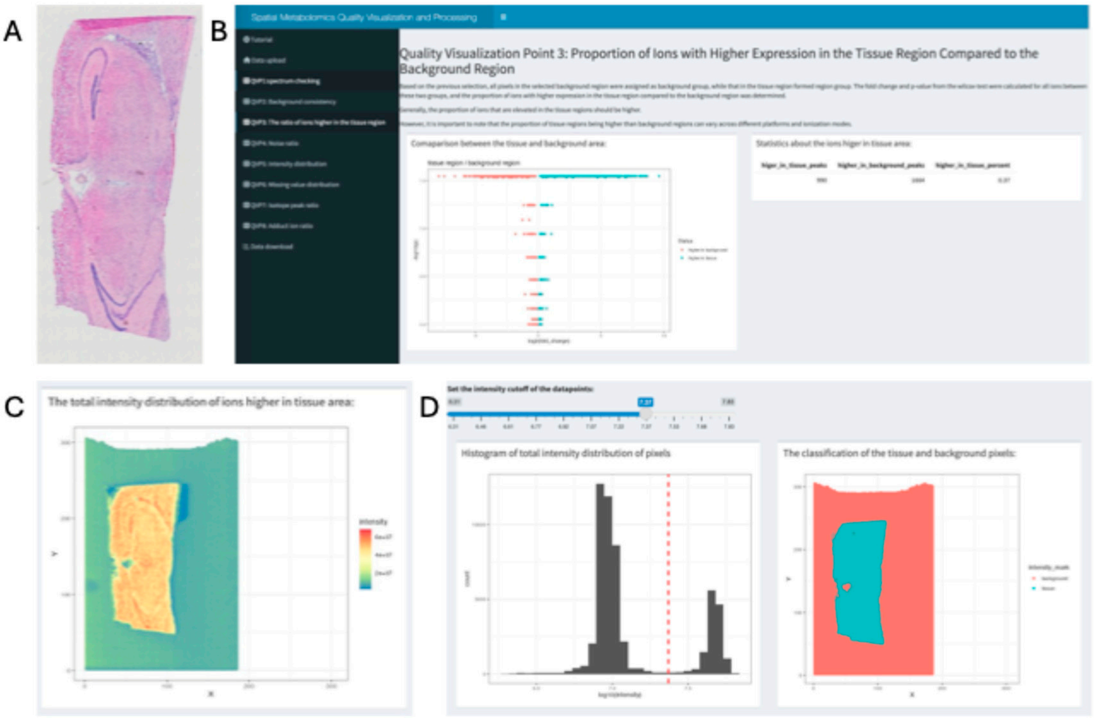

Figure S4. The spatial distribution of ions with the noise scores close to 20.

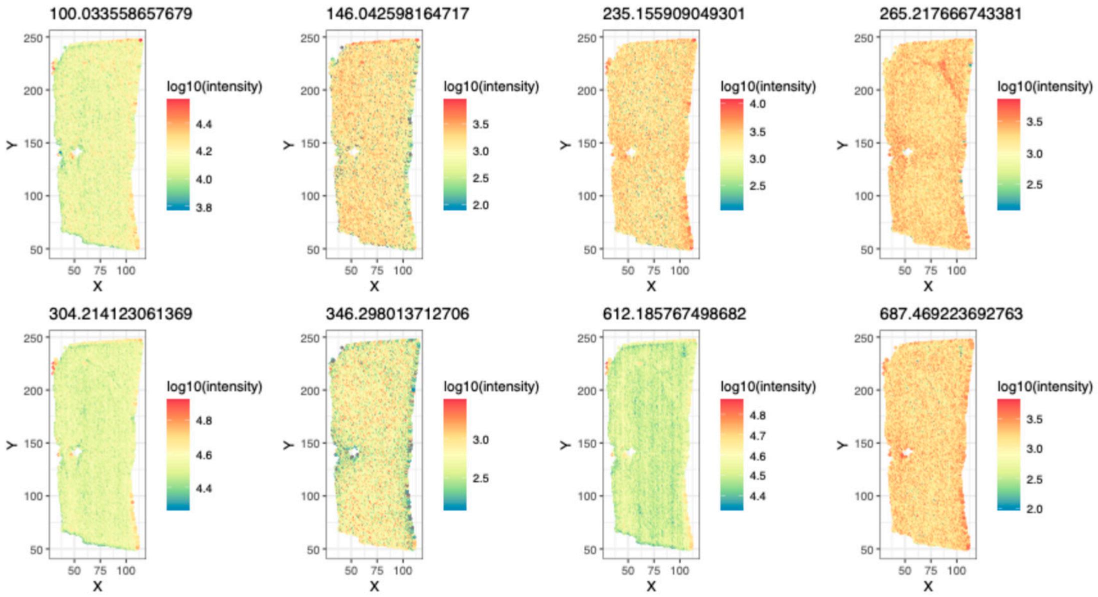

Figure S5. The spatial distribution of ions with the noise scores close to 60.

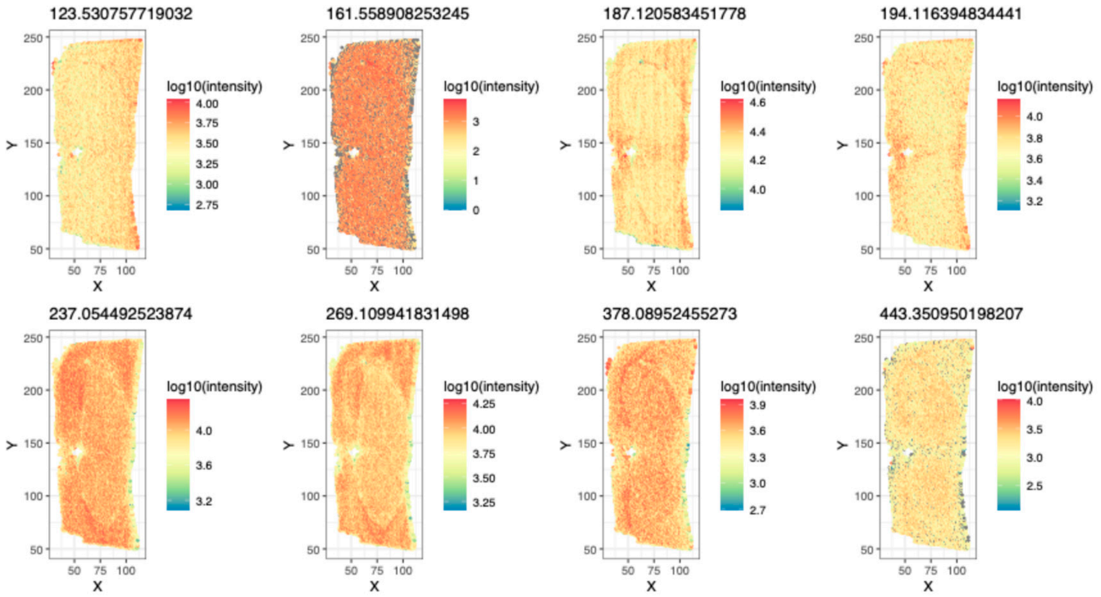

Figure S6. The spatial distribution of ions with the noise scores close to 90.

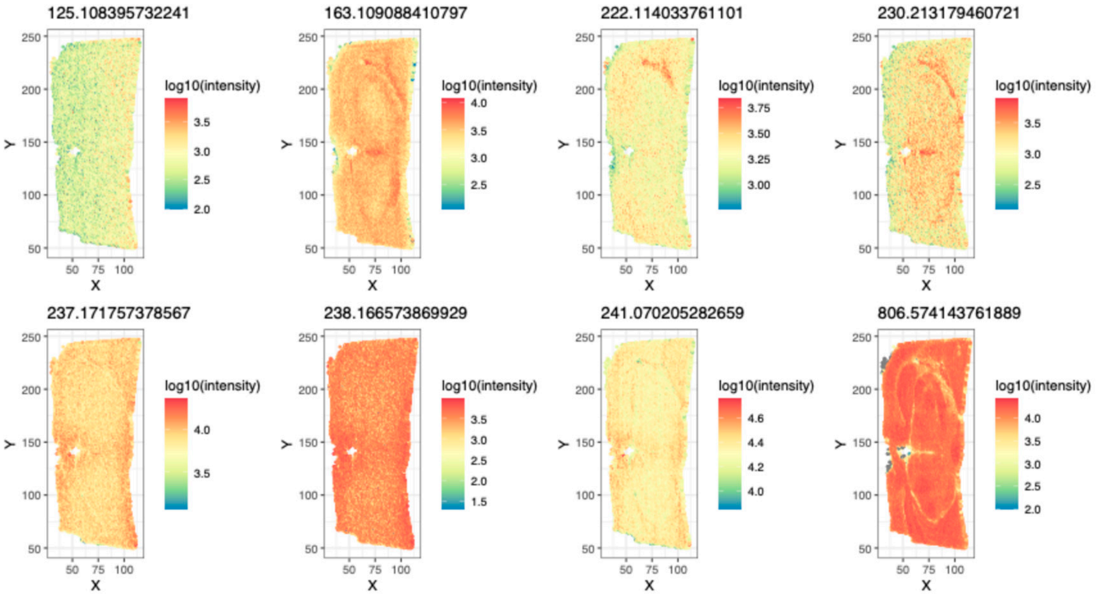

Figure S7. Screenshot depicting the QVP1 visualization of the AP-SMALDI dataset.

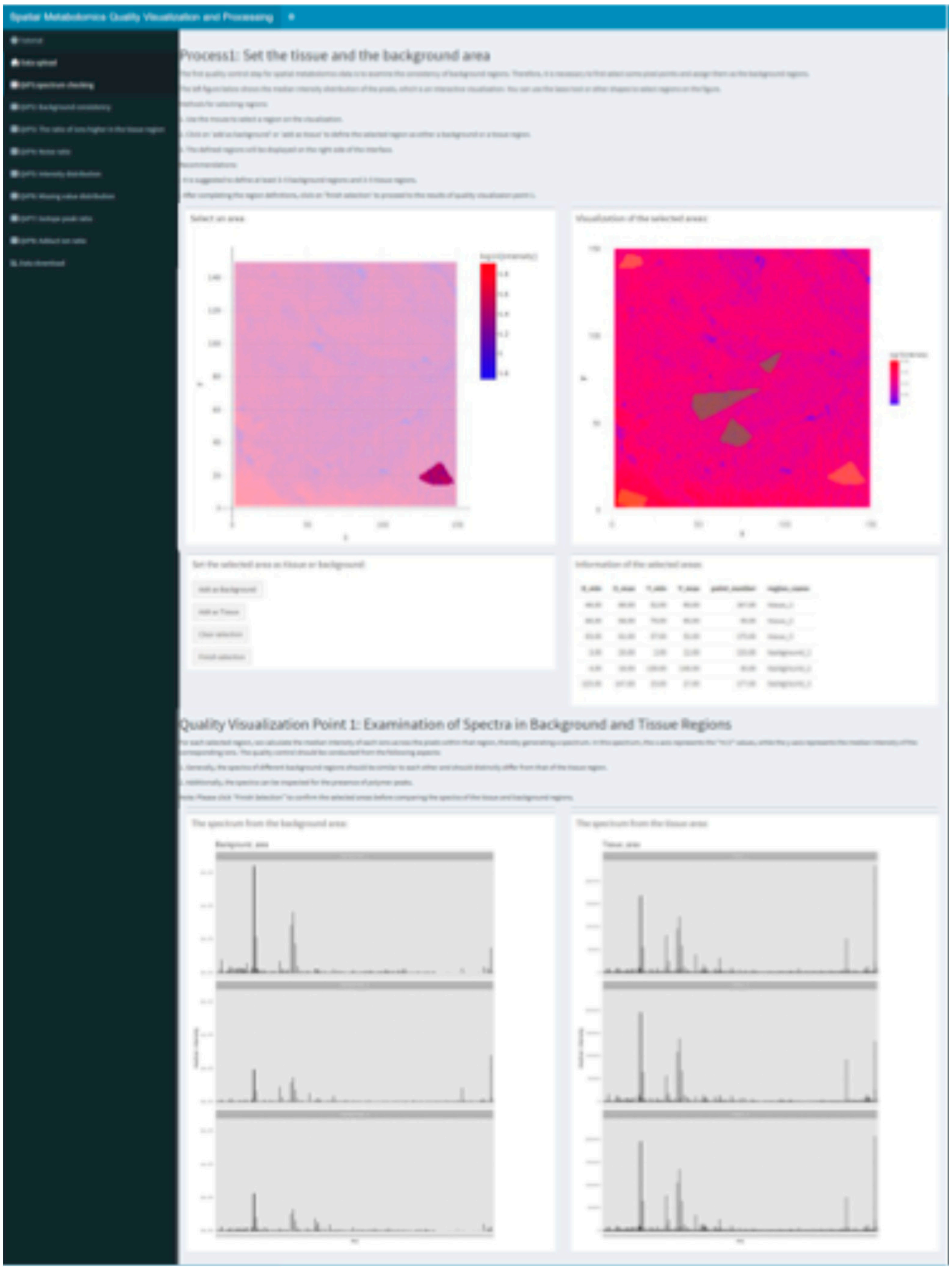

Figure S8. Screenshot depicting the QVP2 visualization of the AP-SMALDI dataset.

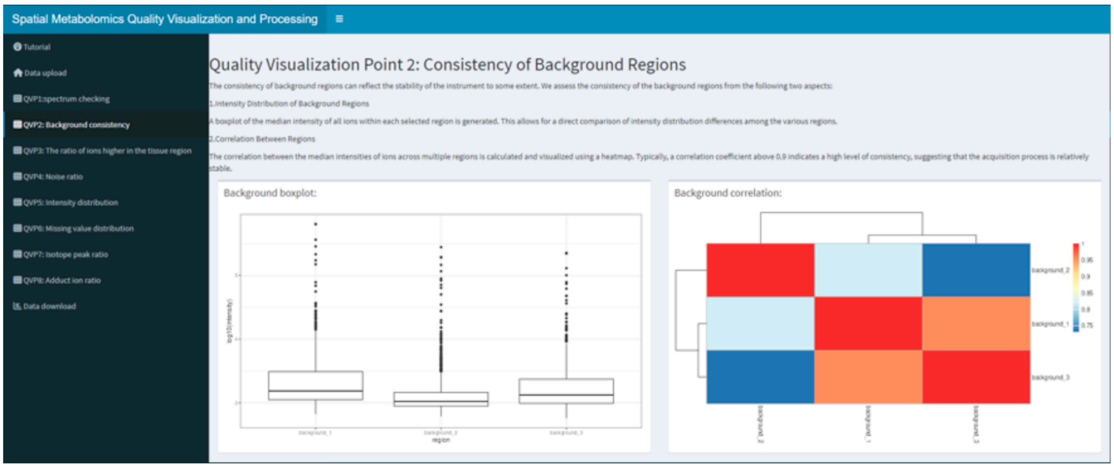

Figure S9. Screenshot depicting the QVP3 visualization of the AP-SMALDI dataset.

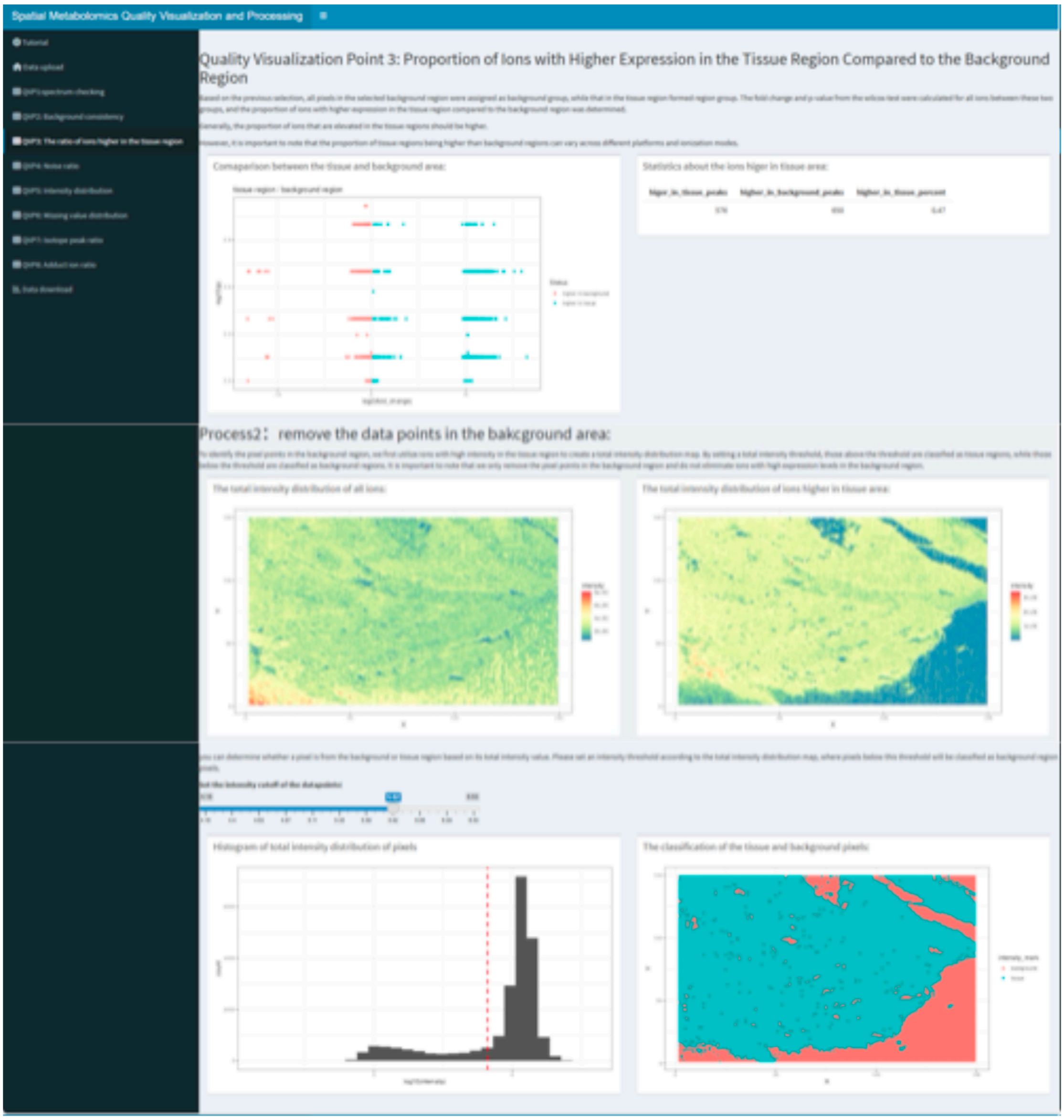

Figure S10. Screenshot depicting the QVP4 visualization of the AP-SMALDI dataset.

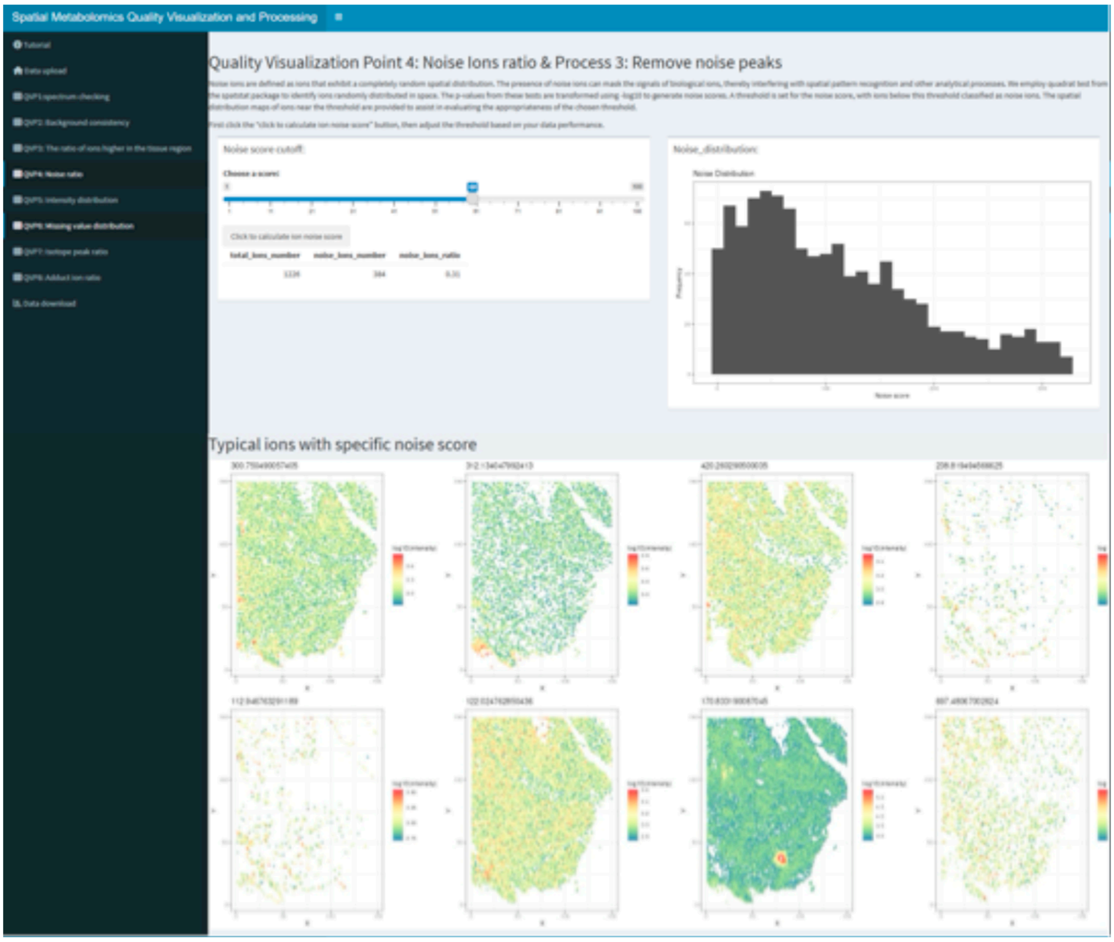

Figure S11. Screenshot depicting the QVP5 visualization of the AP-SMALDI dataset.

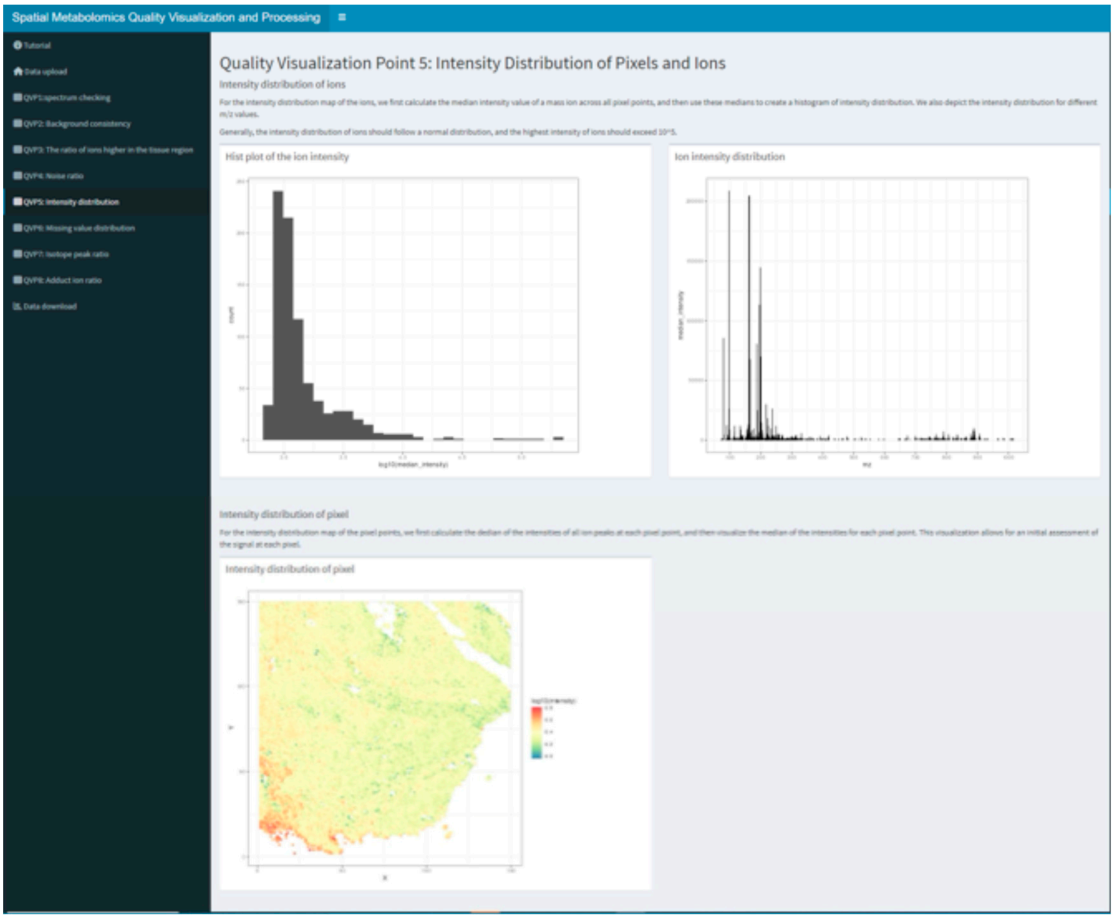

Figure 12. Screenshot depicting the QVP6 visualization of the AP-SMALDI dataset.

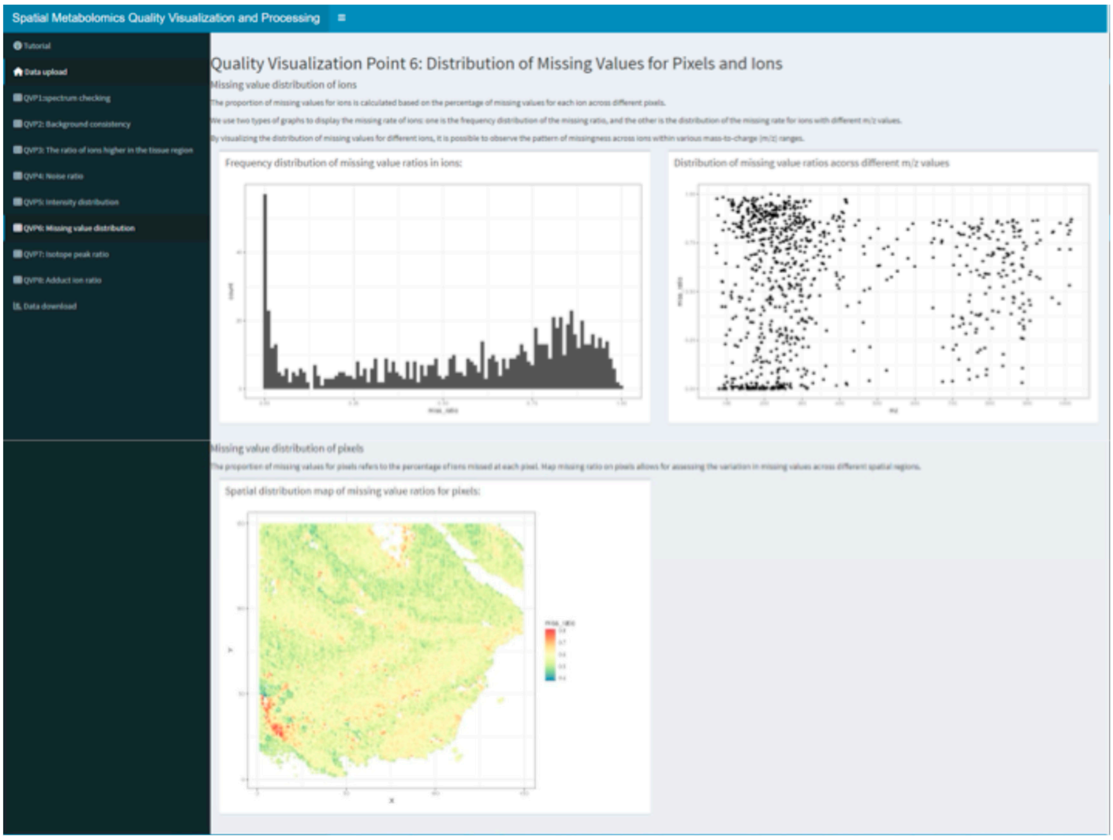

Figure S13. Screenshot depicting the QVP7 visualization of the AP-SMALDI dataset.

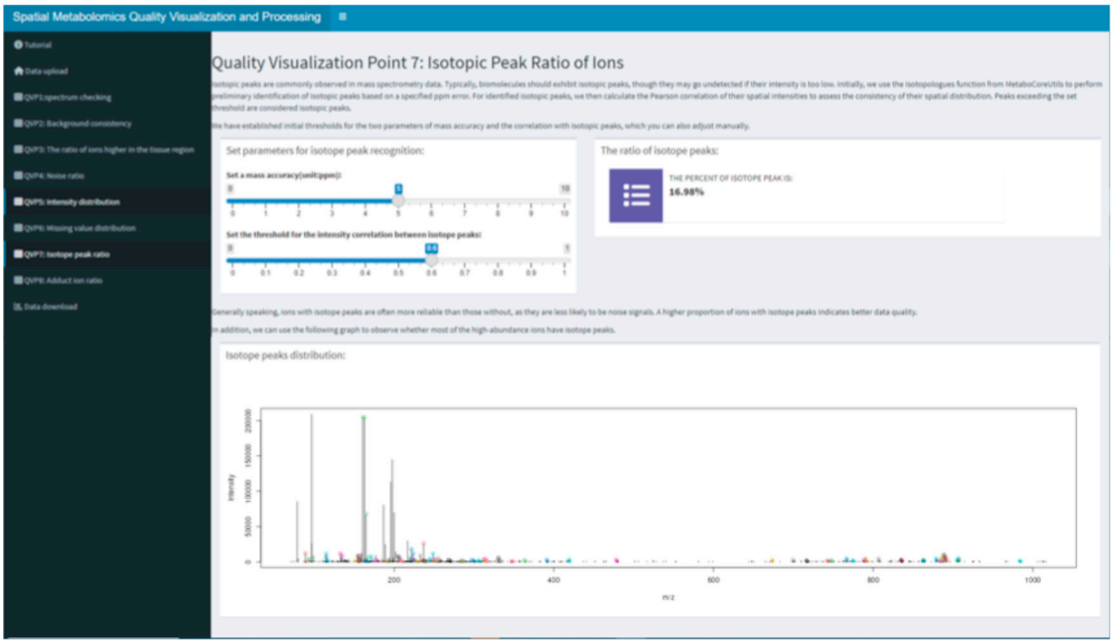

Figure S14. Screenshot depicting the QVP8 visualization of the AP-SMALDI dataset.

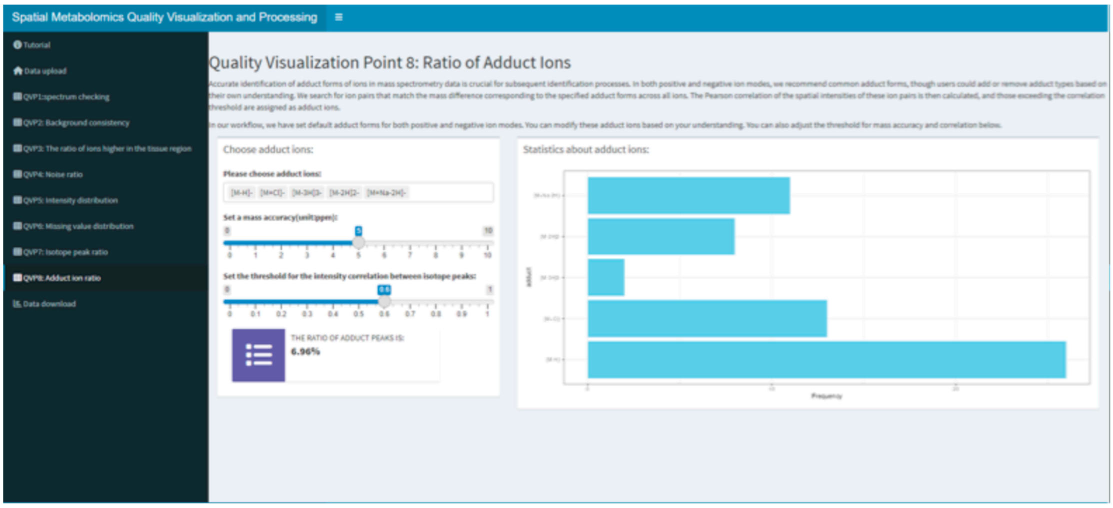

Supplement: Supplementary file 1 [file metabolites-15-00354-s001.zip › metabolites-3639782-supplementary.pdf]
